# Combined use of anticoagulant and antiplatelet on outcome after stroke in patients with nonvalvular atrial fibrillation and systemic atherosclerosis

**DOI:** 10.1038/s41598-023-51013-3

**Published:** 2024-01-03

**Authors:** JoonNyung Heo, Hyungwoo Lee, Il Hyung Lee, In Hwan Lim, Soon-Ho Hong, Joonggyeong Shin, Hyo Suk Nam, Young Dae Kim

**Affiliations:** 1 Department of Neurology, Yonsei University College of Medicine, Seoul, South Korea; 2 Department of Radiology, Yonsei University College of Medicine, Seoul, South Korea

**Keywords:** Cardiology, Neurology, Stroke

## Abstract

This study aimed to investigate whether there was a difference in one-year outcome after stroke between patients treated with antiplatelet and anticoagulation (OAC + antiplatelet) and those with anticoagulation only (OAC), when comorbid atherosclerotic disease was present with non-valvular atrial fibrillation (NVAF). This was a retrospective study using a prospective cohort of consecutive patients with ischemic stroke. Patients with NVAF and comorbid atherosclerotic disease were assigned to the OAC + antiplatelet or OAC group based on discharge medication. All-cause mortality, recurrent ischemic stroke, hemorrhagic stroke, myocardial infarction, and bleeding events within 1 year after the index stroke were compared. Of the 445 patients included in this study, 149 (33.5%) were treated with OAC + antiplatelet. There were no significant differences in all outcomes between groups. After inverse probability of treatment weighting, OAC + antiplatelet was associated with a lower risk of all-cause mortality (hazard ratio 0.48; 95% confidence interval 0.23–0.98; P = 0.045) and myocardial infarction (0% vs. 3.0%, P < 0.001). The risk of hemorrhagic stroke was not significantly different (P = 0.123). OAC + antiplatelet was associated with a decreased risk of all-cause mortality and myocardial infarction but an increased risk of ischemic stroke among patients with NVAF and systemic atherosclerotic diseases.

## Introduction

Nonvalvular atrial fibrillation (NVAF) is the most common cardioembolic source of ischemic stroke^1^. It substantially increases the risk of the stroke, and the outcome after stroke caused by NVAF is worse than any other stroke etiology^2^. For preventing systemic embolism including ischemic stroke, the use of an oral anticoagulant (OAC) such as vitamin K antagonists (VKA) or direct oral anticoagulants (DOAC) is strongly recommended for patients with NVAF^3^.

Systemic atherosclerosis in the brain, heart, and peripheral artery can co-exist in patients with NVAF^4–7^. Furthermore, the co-existence of any systemic atherosclerosis in patients with stroke with NVAF carries an even higher risk of vascular events in the same or different vascular beds, suggesting the need for optimum antithrombotic strategies for these patients^5^. Addition of antiplatelet with OAC could be an option for the management of these patients, while these regimens could lead to an increased risk of bleeding^3,8^. Recent studies suggested that low-dose rivaroxaban combined with aspirin could improve cardiovascular outcomes among patients with coronary arterial occlusive disease (CAOD) or peripheral arterial occlusive diseases (PAOD)^9,10^, which suggests that the use of OAC with antiplatelet might be beneficial for the management of atherosclerotic disease with stroke and NVAF. However, there is limited information on the optimal management of stroke with NVAF and comorbid systemic atherosclerosis, including intracranial or extracranial artery stenosis as well as CAOD or PAOD.

This study aimed to investigate whether the combined use of antiplatelet and OAC could affect outcomes within 1 year after index stroke among patients with NVAF and systemic atherosclerosis.

## Results

### Study population

**Figure 1:**
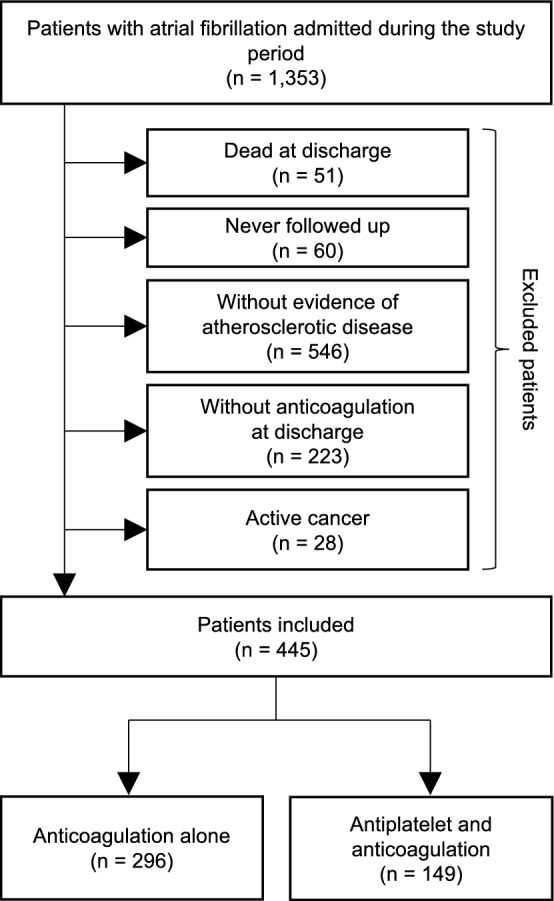
Flow chart of patients included in this study.

Among the 6912 patients with ischemic stroke or TIA, 1353 patients who were previously or newly diagnosed with NVAF were selected. After excluding 51 (3.8%) patients being dead during hospitalization, 60 (4.4%) for loss of follow-up, 546 (40.3%) for not having any evidence of atherosclerotic disease, 223 (16.5%) for not having anticoagulation prescribed, and 28 (2.0%) for active cancer, 445 patients were included in this study (Fig. 1).

### Baseline characteristics

**Figure 2:**
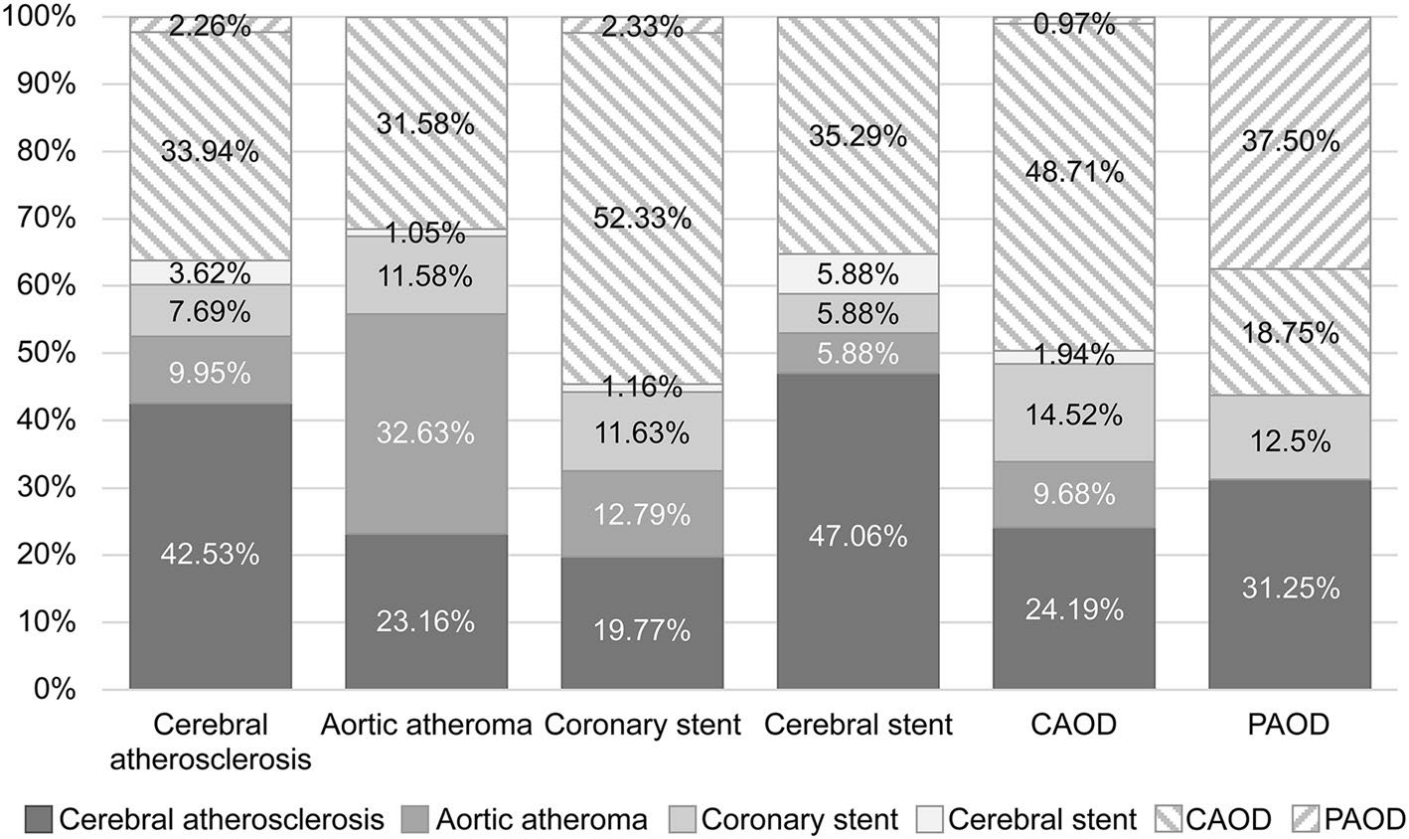
Distribution of patients for each atherosclerotic disease. *CAOD* coronary artery occlusive disease, *PAOD* peripheral artery occlusive disease.

The median age was 75 (interquartile range, IQR 70.0–80.0) and 59.3% (n = 264) was male in the included 445 patients. Of these patients, 193 (43.4%) had cerebral atherosclerosis, 278 (62.5%) had CAOD, 13 (2.9%) had PAOD, 74 (16.6%) had aortic arch atheroma, 62 (13.9%) had coronary stents, and 12 (2.7%) had cerebral artery stents. There were 293 (65.8%) patients with atherosclerotic lesions in one arterial bed, 121 (27.2%) in two beds, 27 (6.1%) in three beds, and four (0.9%) in four beds (Fig. 2 and Supplementary Table 1).

There were 296 (66.5%) patients on OAC alone and 149 (33.5%) on OAC + antiplatelet. Among 296 patients treated with OAC alone, 103 (34.8%) received VKA and 193 (65.2%) received DOAC. Among the 149 patients who received OAC + antiplatelet, 96.6% (n = 144) received a single antiplatelet agent. Aspirin was the most frequently prescribed antiplatelet (n = 65, 43.6%), followed by clopidogrel (n = 63, 42.3%), triflusal (n = 11, 7.4%), cilostazol (n = 5, 3.4%), and a combination of the two antiplatelets (n = 5, 3.4%). VKA was prescribed more frequently than DOAC for patients in the OAC + antiplatelet group (55.0% [82/149] for VKA and 45.0% [67/149] for DOAC). Among the patients who received VKA, there were no differences between international normalized ratio at discharge between the two groups (2.3 [IQR 1.9–2.7] for OAC alone group and 2.4 [IQR 1.9–2.8] for OAC + antiplatelet group, P = 0.585). Among the 260 patients who received DOAC, insufficient dose of DOAC was used in 10% (n = 26) and the proportion of patients who received insufficient dose was not different between the two groups (10.4% [20/193] for OAC alone group and 9.0% [6/67] for OAC + antiplatelet group, P = 0.925). Statin was prescribed at discharge in 97.3% (n = 433). In addition, the statin treatment was not different between the two groups (98.0% [290/296] for OAC only group and 96.0% [143/149] for OAC + antiplatelet group, P = 0.358). There were no significant differences of stroke subtypes based on Trial of Org 10,172 in Acute Stroke Treatment between the two study groups (P = 0.137, Supplementary Table 2). More than two causes subtype was the most common etiology for both groups. Transesophageal echocardiography was performed for 63.4% (n = 282) of the patients, which was not different between the two groups (65.2% [193/296] for OAC only group and 59.7% [89/149] for OAC + antiplatelet group, P = 0.305). When we assessed the maintenance of the antithrombotic regimen using medical chart review and/or electronic prescription code for drugs for 38 patients, whose prescription information could be checked at the time of death during follow-up, the antithrombotic regimen at discharge was maintained for 81.6% (n = 31) of patients.

Compared with OAC alone, patients receiving OAC + antiplatelet were more likely to have hypertension, CAOD, and cerebral and coronary stents (Table 1). Multivariable logistic regression analysis adjusting these four variables showed that CAOD (odds ratio [OR] 1.71; 95% confidence interval [CI] 1.09–2.69; P = 0.019), cerebral stent (OR 5.31; 95% CI 1.51–18.60; P = 0.009), and coronary stent (OR 6.24; 95% CI 3.42–11.39; P < 0.001) were independent determinants for the use of OAC + antiplatelet.

**Table 1:** Comparison of baseline characteristics between patients who received a combination of antiplatelet and anticoagulation and anticoagulation alone**.**

	Unadjusted groups			Adjusted groups		
	OAC + Antiplatelet (n = 149)	OAC (n = 296)	D (%)	OAC + Antiplatelet (n = 152.2)	OAC (n = 284.5)	D (%)
Age (> 75 years)	76 (51.0)	131 (44.3)	13.5	67.0 (44.0)	127.7 (44.9)	1.7
Sex (Male)	94 (63.1)	170 (57.4)	11.5	86.1 (56.5)	167.7 (59.0)	4.9
Hypertension	135 (90.6)	246 (83.1)	22.3	128.3 (84.3)	243.1 (85.5)	3.3
Diabetes	60 (40.3)	105 (35.5)	9.9	57.8 (38.0)	109.4 (38.4)	0.9
Dyslipidemia	65 (22.0)	33 (22.1)	0.5	37.8 (24.8)	63.9 (22.5)	5.6
CAOD	106 (71.1)	172 (58.1)	27.4	94.3 (62.)	178.2 (62.6)	1.3
PAOD	3 (2.0)	10 (3.4)	8.4	2.6 (1.7)	5.6 (2.0)	2.0
Current smoker	22 (14.8)	38 (12.8)	5.6	18.0 (11.8)	36.3 (12.8)	2.8
CHF	17 (11.4)	20 (6.8)	16.2	13.0 (8.5)	24.1 (8.5)	0.2
Cerebral stent	8 (5.4)	4 (1.4)	22.4	3.3 (2.2)	5.4 (1.9)	1.9
Coronary stent	44 (29.5)	18 (6.1)	64.2	19.9 (13.1)	33.7 (11.9)	3.7
Initial NIHSS (> 4)	74 (49.7)	146 (49.3)	0.7	77.5 (50.9)	141.1 (49.6)	2.7
Low GFR	6 (4.0)	7 (2.4)	9.4	4.9 (3.2)	10.3 (3.6)	2.3
Cerebral artery stenosis	70 (47.0)	123 (41.6)	10.9	67.2 (44.1)	120.6 (42.4)	3.5
Aortic atheroma	24 (16.1)	50 (16.9)	2.1	22.2 (14.6)	42.9 (15.1)	1.4
High dose statin	102 (68.5)	226 (76.4)	17.7	111.8 (73.4)	212.3 (74.6)	2.6

Values are presented as number (%) for categorical variables and median [interquartile range] for continuous variables.

*CAOD* coronary artery occlusive disease, *PAOD* peripheral artery occlusive disease, *CHF* chronic heart failure, *NIHSS* National Institutes of Health stroke scale, *GFR* glomerular filtration rate.

### Comparison of outcomes in the original study population

During the 1-year follow-up (median, 365 days; IQR 321–365 days), all-composite outcome within 1 year occurred in 93 (20.9%) patients. For each outcome, there were all-cause mortalities in 50 (11.2%), recurrent ischemic stroke in 16 (2.9%) (3 [0.7%] fatal and 13 [2.9%] non-fatal), myocardial infarction in five (1.1%) (3 [0.7%] fatal and 2 [0.4%] non-fatal), hemorrhagic stroke in four (0.9%, all non-fatal), and bleeding events in 35 (7.9%) (1 [0.2%] fatal and 34 [7.6%] non-fatal) patients. There was no difference in the rates of all predetermined outcomes between antithrombotic regimens (Table 2).

**Table 2:** Hazard ratios for the prescription of combination of oral anticoagulant with antiplatelet on outcomes.

	Unadjusted groups				Adjusted groups			
	OAC + Antiplatelet (n = 149)	OAC (n = 296)	Hazard ratio	P-value	OAC + Antiplatelet (n = 152.2)	OAC (n = 284.5)	Hazard ratio	P-value
All-composite outcome	31 (20.8)	62 (20.9)	0.93 [0.60;1.45]	0.750	29.6 (19.4)	60.2 (21.1)	0.91 [0.55;1.50]	0.709
All-cause mortality	13 (8.7)	37 (12.5)	0.63 [0.33;1.20]	0.159	9.3 (6.1)	34.6 (12.2)	0.48 [0.23;0.98]	0.045
Cardiovascular death	4 (2.7)	13 (4.4)	0.60 [0.19;1.83]	0.365	2.3 (1.5)	12.1 (4.3)	0.34 [0.10;1.17]	0.087
Ischemic stroke (fatal, nonfatal)	10 (6.7)	9 (3.0)	2.19 [0.89;5.38]	0.089	13.5 (8.8)	8.9 (3.1)	2.76 [1.03;7.40]	0.044
Myocardial infarction (fatal, nonfatal)	0 (0.0)	7 (2.4)	0.00 [0.00;Inf]	0.998	0.0 (0.0)	8.4 (3.0)	0.00 [0.00;0.00]	< 0.001
Hemorrhagic stroke (fatal, nonfatal)	3 (2.0)	1 (0.3)	5.99 [0.62;57.60]	0.121	3.8 (2.5)	1.2 (0.4)	6.12 [0.61;60.91]	0.123
Bleeding (fatal, nonfatal)	13 (8.7)	21 (7.1)	1.12 [0.55;2.27]	0.760	11.9 (7.8)	21.4 (7.5)	1.01 [0.46;2.21]	0.982

Values are represented as numbers (%) for incidence and hazard ratios [95% confidence intervals]. Only the first occurrence of each outcome during the 1 year follow-up period was included.

*Inf* infinite, *MI* myocardial infarction.

### Comparison of outcomes in the matched population

**Figure 3:**
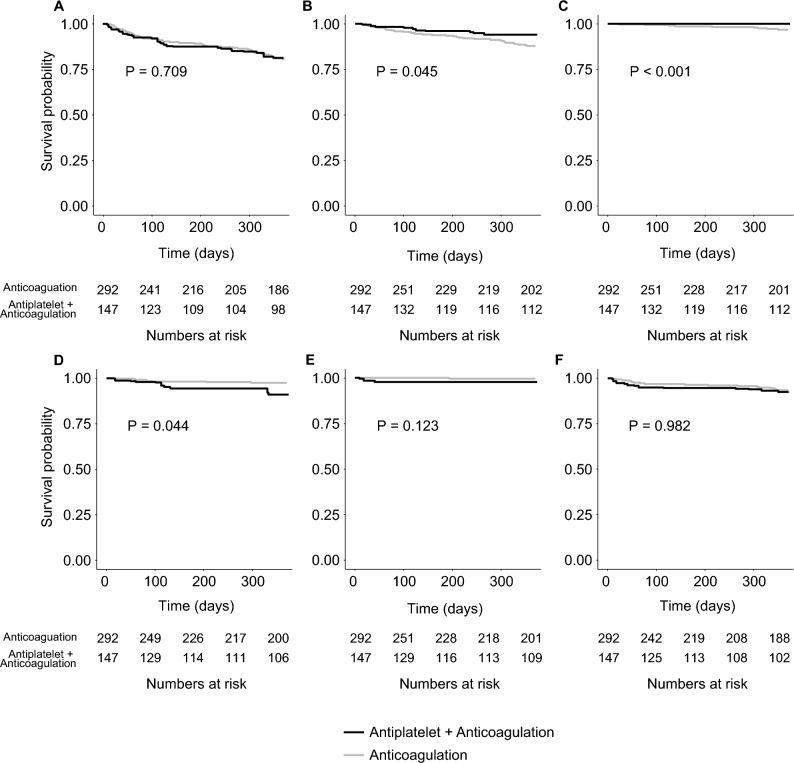
Kaplan–Meier survival curves of (**A**) all-composite outcome, (**B**) all-cause mortality, (**C**) myocardial infarction, (**D**) ischemic stroke, (**E**) hemorrhagic stroke, and (**F**) bleeding event by antithrombotic regimens in the matched population.

**Figure 4:**
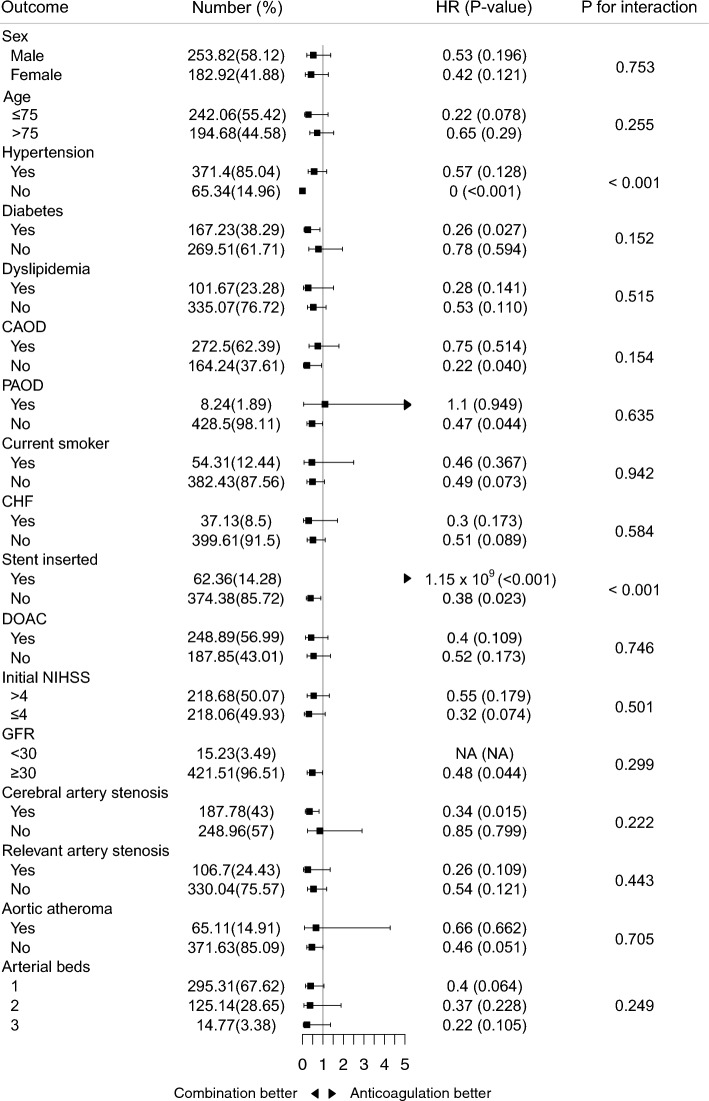
Subgroup analysis of patients weighted with inverse probability of treatment weighting. Cox proportional hazard ratios were obtained for each subgroup.Combination indicates combination of antiplatelet and anticoagulation. The arterial bed count was defined as the number of atherosclerotic diseases a patient had, which included cerebral artery stenosis, CAOD, PAOD, aortic atheroma, and coronary and cerebral stent insertion history. *CAOD* coronary artery occlusive disease, *PAOD* peripheral artery occlusive disease, *CHF* chronic heart failure, *DOAC* direct oral anticoagulants, *NIHSS* national institutes of health stroke scale, *GFR* glomerular filtration rate, *HR* hazard ratio.

After performing inverse probability of treatment weighting, the baseline characteristics were well balanced with weighted sample sizes of 284.5 for the OAC group and 152.2 for OAC + antiplatelet group (Table 1 and Supplementary Fig. 1). The Kaplan–Meier estimate showed that the all-composite outcome did not differ according to antithrombotic regimen. However, OAC alone had a higher risk of death within 1 year compared to OAC + antiplatelet (log-rank P = 0.045, Fig. 3). Cox regression analysis showed that all-cause mortality within 1 year was significantly higher in patients who received OAC alone than in those who received OAC + antiplatelet (6.1% vs. 12.2%; hazard ratio, 0.48; 95% CI 0.23–0.98; P = 0.045). In the subgroup analysis, the effect of OAC + antiplatelet treatment on all-cause mortality was overall consistent regardless of baseline characteristics, while there were interactions between antithrombotic type and cerebral stent, coronary stent, or hypertension (Fig. 4). The use of OAC + antiplatelet was also associated with a lower risk of cardiovascular death (1.5% vs. 4.3%, P = 0.087) or myocardial infarction (0% vs. 0.6%, P < 0.001). However, recurrent ischemic stroke was more common in patients who received OAC + antiplatelet (8.8% vs. 3.1%; hazard ratio, 2.76; 95% CI 1.03–7.40; P = 0.044) (Table 2), and most recurrent ischemic strokes were non-fatal (Supplementary Table 3). Other predetermined outcomes, such as hemorrhagic stroke and bleeding events, did not differ according to antithrombotic regimen.

## Discussion

This study investigated whether one-year outcome could differ according to antithrombotic strategy after index ischemic stroke among patients with NVAF and systemic atherosclerosis. Although the combined use of OAC and antiplatelet therapy had no beneficial effect in the original population and there was no difference in all-composite outcomes in the matched population, inverse probability of treatment weighting analysis showed that the risk of all-cause mortality or myocardial infarction was lower in patients with combined use of OAC and antiplatelet when patients had NVAF and systemic atherosclerosis. These trends were consistently observed in various subgroups, except in patients with cerebral or coronary stents or hypertension. However, the risk of recurrent ischemic stroke was higher in patients receiving antiplatelet therapy along with OAC.

Comorbid atherosclerosis is not rare among patients with NVAF. Co-existing atherosclerotic diseases can be observed in either the carotid artery (up to 64%)^11^, coronary artery (17–38%)^12^, or peripheral artery disease (6.7%)^12^. In this study, CAOD was the most prevalent site of atherosclerosis (63%) followed by cerebral atherosclerosis (43%). Furthermore, approximately one-third of the patients had atherosclerotic disease with two or more arterial beds. In addition, if any atherosclerotic diseases are present, cerebro-cardiovascular outcomes could be worse among patients with atrial fibrillation^5,13,14^. Therefore, there are concerns regarding the management of patients with NVAF and systemic atherosclerosis.

Considering the higher risk of cardiovascular events among patients with NVAF with coexisting atherosclerosis, a more potent therapeutic strategy is required. Currently, data on the optimal antithrombotic strategy for this specific population are limited. Because antiplatelets can inhibit platelet aggregation and prevent thrombus formation under conditions of elevated shear stress and high velocity, adding antiplatelet to OAC could be beneficial for patients with atherothrombotic diseases. However, the combined use of antiplatelet and OAC for patients with NVAF is challenging because previous retrospective studies and subsequent meta-analyses demonstrated that the benefit of antiplatelet with OAC for preventing ischemic events was not clearly observed or only noted in a specific situation^8^. In our study, the beneficial effect of the combined use of OAC and antiplatelet was not observed in the original study population, whereas the benefit of these regimens was noted in terms of all-cause mortality and myocardial infarction after balancing the baseline characteristics using inverse probability of treatment weighting. This suggests that patient characteristics or comorbidities may affect the efficacy of medication, and an optimum selection of patients who are supposed to benefit from OAC + antiplatelet would be necessary. In this context, further investigation on the population that would benefit from the combined use of OAC and antiplatelet is warranted.

However, ischemic stroke recurrence was higher for those who were prescribed OAC + antiplatelet, although most recurrent ischemic strokes on OAC + antiplatelet therapy during follow-up did not seem to be severe or fatal in our study. This implies that the combined use of an antithrombotic regimen would be less effective for the prevention of recurrent ischemic stroke than the use of OAC alone. The stroke risk and severity in patients with NVAF is affected by the intensity of VKA or dosage of DOAC^15,16^. Physicians might be reluctant to use the optimal dose of DOAC or to maintain the therapeutic intensity of VKA during follow-up because of concerns regarding bleeding risk, although we could not investigate the intensity of OAC during follow-up. Furthermore, considering that the risk of cardiovascular events increases with an increase in the burden of cerebral stenosis^17^, the addition of antiplatelet with OAC might not be enough to prevent recurrent ischemic stroke. These findings suggest that a more potent therapeutic approach is required to prevent recurrent strokes in this population. For example, vascular risk factors such as hypertension, dyslipidemia, and diabetes are well-known determinants of thromboembolism caused by atrial fibrillation and atherosclerotic diseases^18–20^, and strict control of vascular risk factors during follow-up would be helpful.

When using OAC with antiplatelet, concerns on increase in the risk of bleeding complication are raised^21^. Concurrent use of antiplatelet with OAC increased the bleeding risk (especially at gastrointestinal or intracranial site) compared to OAC alone^8^. However, in our study, even though cerebral hemorrhages seemed to be frequent in case of OAC + antiplatelet, there was no significant difference in bleeding complications between OAC + antiplatelet and OAC only. Bleeding complications were less likely to occur when using DOAC with antiplatelet, rather than VKA with antiplatelet^22^ or using OAC with single rather than OAC with dual antiplatelet^23^. In our patients using OAC + antiplatelet, a single antiplatelet agent was mostly used and many patients were treated with DOAC. In addition, strict vascular risk factor control during follow-up was regularly monitored at our stroke center. These might be related to our findings.

Our study has several limitations. In this study, we divided the study population according to the medication on discharge. Although the medication during follow-up seemed to be well maintained after discharge, we could not assess the compliance or intensity of antithrombotic agents completely. Even though inverse probability of treatment weighting, a validated statistical method to assess the effect of different treatments using retrospective data^24^, was performed to eliminate selection bias for antithrombotic therapy, there could be other factors that we did not consider. We only investigated 1 year outcomes after index stroke; therefore, this result did not imply any benefit of long-term use of OAC + antiplatelet. Considering that the dataset was from a single center, as well as the retrospective nature of the study, there could be selection bias in the dataset. Randomized controlled studies should be performed to eliminate selection bias between the two groups.

## Methods

### Patients

This was a retrospective study using data from a hospital-based observational cohort study (Clinicaltrials.gov NCT03510312) investigating the long-term prognosis of patients with ischemic stroke or transient ischemic attack (TIA) who were admitted to the Severance Stroke Center located in Seoul, Republic of Korea. This cohort included patients with acute cerebral infarction or TIA within 7 days of onset who were admitted between January 1, 2012, and December 31, 2020. All patients underwent brain CT and/or magnetic resonance imaging along with angiography, including CT angiography, magnetic resonance angiography, or digital subtraction angiography. During hospitalization, all the patients were extensively and thoroughly evaluated to determine their demographic data, medical history, clinical manifestations, vascular risk factors, and comorbidities. The patients also underwent standard blood tests, 12-lead electrocardiography, and Holter monitoring. Nearly all patients were admitted to the stroke unit and monitored continuously using electrocardiography during their stay (median, 5 days; IQR 4–6 days). Echocardiography, including transesophageal echocardiography, was a routine examination unless it could not be performed because of the patient’s condition or failure to obtain informed consent. This study was approved by the Institutional Review Board of Yonsei University College of Medicine (approval number: 4-2022-1098) with a waiver of informed consent owing to the retrospective nature of the study. All methods in our study were performed in accordance with the tenets of the Declaration of Helsinki.

### Clinical variables

For each patient, demographic data, vascular risk factors, and comorbidities including hypertension, diabetes mellitus, dyslipidemia, CAOD, PAOD, and history of stroke were collected. Current smoking (one or more cigarettes smoked during the last month) and alcohol consumption status were also assessed. Stroke severity was assessed using the National Institutes of Health Stroke Scale (NIHSS). Premorbid disability was defined as a modified Rankin Scale (mRS) score of 2–5 before the index stroke. Stroke subtypes were classified according to the Trial of Org 10172 in the Acute Stroke Treatment classification^25^. Estimated glomerular filtration was calculated using the CKD-EPI method. Data on statin medications prescribed upon discharge were also collected. High-intensity statin therapy was defined as atorvastatin > 40 mg or rosuvastatin > 20 mg^26^.

Our study population was stratified according to the antithrombotic regimen prescribed upon discharge: OAC alone versus combined OAC and antiplatelet. OAC consisted of VKA and DOAC, including dabigatran, rivaroxaban, apixaban, and edoxaban. Antiplatelet agents included aspirin, clopidogrel, cilostazol, triflusal, prasugrel, ticlopidine, and aggrenox.

### Systemic atherosclerotic lesion

The presence of atherosclerotic lesions in the arteries of the aorta, brain, heart, and peripheral arteries was investigated in each patient. This included one or more of the following atherosclerotic diseases: (1) cerebral atherosclerosis with > 50% stenosis based on the North American Symptomatic Carotid Endarterectomy Trial method^27^ for extracranial artery or Warfarin vs. Aspirin for Symptomatic Intracranial Disease method^28^ for intracranial artery; (2) > 4 mm aortic atheroma at the ascending aorta or aortic arch on transesophageal echocardiography; (3) previous history of CAOD or PAOD; and (4) coronary or carotid stent insertion. To prevent the misinterpretation of embolic occlusion as cerebral atherosclerosis, we included only stenotic lesions after excluding arterial culprit lesions in infarcted areas or complete occlusion of the cerebral artery. CAOD was defined as (1) previous history of myocardial infarction or unstable angina and (2) coronary artery stenosis, either symptomatic or asymptomatic, on documented catheter coronary angiography or multidetector coronary CT. The presence of PAOD was defined when it was verified by sonographic evaluation or CT angiography. The burden of systemic atherosclerosis was calculated as the total number of atherosclerotic diseases including cerebral artery stenosis, CAOD, PAOD, aortic atheroma, and coronary or cerebral stent insertion history.

### Outcome measures

In our stroke center, stroke neurologists and research nurses regularly contacted patients or their caregivers via regular face-to-face visits or telephone interviews with or without medical chart review to investigate the development of vascular events, cancer, or risk factors newly detected during follow-up. Patients included in this study were regularly followed-up at 3 months and 1 year after discharge.

When a patient died, we recorded the date and if possible, the cause of death was determined. Cardiovascular death was defined as death resulting from myocardial infarction, sudden cardiac death, heart failure, cardiovascular procedure, or stroke^29^. We also collected the data on the occurrence of recurrent ischemic stroke (including TIA), hemorrhagic stroke, myocardial infarction, or bleeding events within 1 year after stroke and if any, the date of events. Recurrent ischemic stroke was determined by a stroke specialist based on the clinical and radiological findings. Hemorrhagic stroke included intracerebral, intraventricular, subarachnoid, and subdural hemorrhages. Myocardial infarction was defined as the diagnosis of acute myocardial infarction based on clinical symptoms, cardiac enzymes, electrocardiography, and imaging studies, such as coronary angiography. All bleeding events were recorded, including hemorrhagic stroke, gastrointestinal bleeding, respiratory bleeding, and muscular bleeding requiring transfusion or hospitalization. Fatal ischemic stroke, fatal hemorrhagic stroke, fatal myocardial infarction, and fatal bleeding events were defined as death after severe respective disease without any other obvious cause of death, which was decided by the physician^16,28^. The primary outcome of this study was the all-composite outcome within 1 year of the index stroke. We compared these predetermined outcomes within 1 year of the index stroke according to medication.

### Statistical analysis

The baseline characteristics of the study participants were compared. Continuous variables were compared using the Mann–Whitney *U* test, and categorical variables using Pearson’s chi-square test. To find out independent factor for the use of antiplatelet along with OAC, variables significantly associated with OAC + antiplatelet group was selected. Using these variables, multivariable logistic regression analysis was performed to finally derive the odds ratio for each variable.

To adjust for substantial differences in baseline characteristics between the OAC alone and OAC + antiplatelet groups, inverse probability of treatment weighting was performed. Propensity scores were estimated for all the patients enrolled in this study using logistic regression. The selected confounders were older age (> 75 years), sex, hypertension, diabetes, dyslipidemia, current smoking, congestive heart failure, high initial NIHSS score (> 4), low estimated glomerular filtration rate (< 30 mL/min/1.73 m^2^), presence of aortic atheroma (> 4 mm), CAOD, PAOD, cerebral atherosclerosis with > 50% stenosis, history of coronary stent insertion, history of cerebral artery stent insertion, and prescription of high-dose statin on discharge. The inverse probability of treatment weighting was calculated by weighting each case using inverse propensity score. Symmetrical trimming was performed with an optimal cut-off to achieve the final dataset^30^. Subgroup analysis was performed according to all variables used in this analysis.

A Kaplan–Meier estimate of survival with the log-rank test was used to compare the differences in event rates of outcomes between the OAC alone and OAC + antiplatelet groups. Cox proportional hazard regression was performed to compare the outcomes between the treatment groups. All *P* values were two-sided. R version 4.0 (R Core Team, Vienna, Austria) was used for statistical analysis^31^. Inverse probability of treatment weighting was performed with the “PSWeight” package and survival analysis using the “survival” package^32,33^.

### Supplementary Information

Supplementary Information.

## Data Availability

The data supporting the findings of this study are available from the corresponding author on reasonable request.
