# Supplementary material for: Combined use of anticoagulant and antiplatelet on outcome after stroke in patients with nonvalvular atrial fibrillation and systemic atherosclerosis
Source: Sci Rep. 2024 Jan 3;14:304. doi: 10.1038/s41598-023-51013-3 (PMC10764735; doi:10.1038/s41598-023-51013-3)
Supplement: Supplementary file 1 — Supplementary Information. [file 41598_2023_51013_MOESM1_ESM.pdf]

Supplementary Materials

Combined use of anticoagulant and antiplatelet on outcome after stroke in patients with nonvalvular atrial fibrillation and systemic atherosclerosis

JoonNyung Heo[1,2], Hyungwoo Lee[1,2], Il Hyung Lee[1,2], In Hwan Lim[1], Soon-Ho Hong[1],

Joonggyeong Shin[1], Hyo Suk Nam[1] and Young Dae Kim[1]

[1]Department of Neurology, Yonsei University College of Medicine, Seoul, South Korea

[2]Department of Radiology, Yonsei University College of Medicine, Seoul, South Korea

Corresponding author:

Young Dae Kim, MD, PhD

Department of Neurology, Yonsei University College of Medicine

50-1 Yonsei-ro, Seodaemun-gu, Seoul 03722, Korea

Tel: +82-2-2228-1619

Fax: +82-2-393-0705

E-mail: neuro05@yuhs.ac

List of items

Supplementary Table 1. Distribution of patients with atherosclerotic diseases
Supplementary Table 2. Trial of Org 10172 in Acute Stroke Treatment classification between the study groups.
Supplementary Table 3. Hazard ratios for prescription of a combination of oral anticoagulants and antiplatelet for fatal and non-fatal outcomes.
Supplementary Figure 1. Standardized mean differences between the groups before and after inverse probability of treatment weighting.

Supplementary Table 1. Distribution of patients with atherosclerotic diseases

	Cerebral atherosclerosis	Aortic atheroma	Coronary stent	Cerebral stent	CAOD	PAOD	Total
Cerebral atherosclerosis	94	22	17	8	75	5	193
Aortic atheroma	22	31	11	1	30	0	74
Coronary stent	17	11	10	1	45	2	62
Cerebral stent	8	1	1	1	6	0	12
CAOD	75	30	45	6	151	3	278
PAOD	5	0	2	0	3	6	13
Total	193	74	62	12	278	13	445

Values are presented as number.

CAOD, coronary artery occlusive disease; PAOD, peripheral artery occlusive disease.

Supplementary Table 2. Trial of Org 10172 in Acute Stroke Treatment classification between the study groups.

	OAC + Antiplatelet (n = 149)	OAC (n = 296)	P-value
Stroke mechanisms			0.137
CE	53 (35.6)	133 (44.9)	
CE+LAA	81 (54.4)	124 (41.9)	
CE+LAC	9 (6.0)	18 (6.1)	
CE+LAA+LAC	3 (2.0)	11 (3.7)	
TIA	3 (2.0)	10 (3.4)	

TIA, transient ischemic attack; CE, cardioembolism; LAA, large artery atherosclerosis; LAC, small-vessel occlusion.

.

Supplementary Table 3. Hazard ratios for prescription of a combination of oral anticoagulants and antiplatelet for fatal and non-fatal outcomes.

	Unadjusted Groups				*Adjusted Groups*			
	OAC + Antiplatelet (n=149)	OAC (n=296)	Hazard ratio	P-value	OAC + Antiplatelet (n=152.2)	OAC (n=284.5)	Hazard ratio	P-value
**Fatal stroke**	3 (2.0)	3 (1.0)	1.94 [0.39;9.61]	0.417	1.9 (1.2)	2.7 (1.0)	1.24 [0.22;6.88]	0.805
**Non-fatal stroke**	7 (4.7)	6 (2.0)	2.29 [0.77;6.83]	0.135	11.6 (7.6)	6.2 (2.2)	3.40 [1.06;10.93]	0.040
**Fatal MI**	0 (0.0)	7 (2.4)	0.00 [0.00;Inf]	0.998	0.0 (0.0)	6.6 (2.3)	0.0 [0.0;0.0]	< 0.001
**Non-fatal MI**	0 (0.0)	2 (0.7)	0.00 [0.00;Inf]	0.999	0.0 (0.0)	1.8 (0.6)	0.0 [0.0;0.0]	< 0.001
**Fatal bleeding**	0 (0.0)	2 (0.7)	0.00 [0.00;Inf]	0.999	0.0 (0.0)	2.0 (0.7)	0.0 [0.0;0.0]	< 0.001
**Non-fatal bleeding**	13 (8.7)	20 (6.8)	1.17 [0.57;2.40]	0.663	11.9 (7.8)	20.2 (7.1)	1.07 [0.48;2.36]	0.871

Values are represented as numbers (%) for incidence and hazard ratios [95% confidence intervals]. Only the first occurrence of each outcome during the 1-year follow-up period was included.

Inf, infinite; MI, myocardial infarction.

Supplementary Figure 1. Standardized mean differences between the groups before and after inverse probability of treatment weighting.

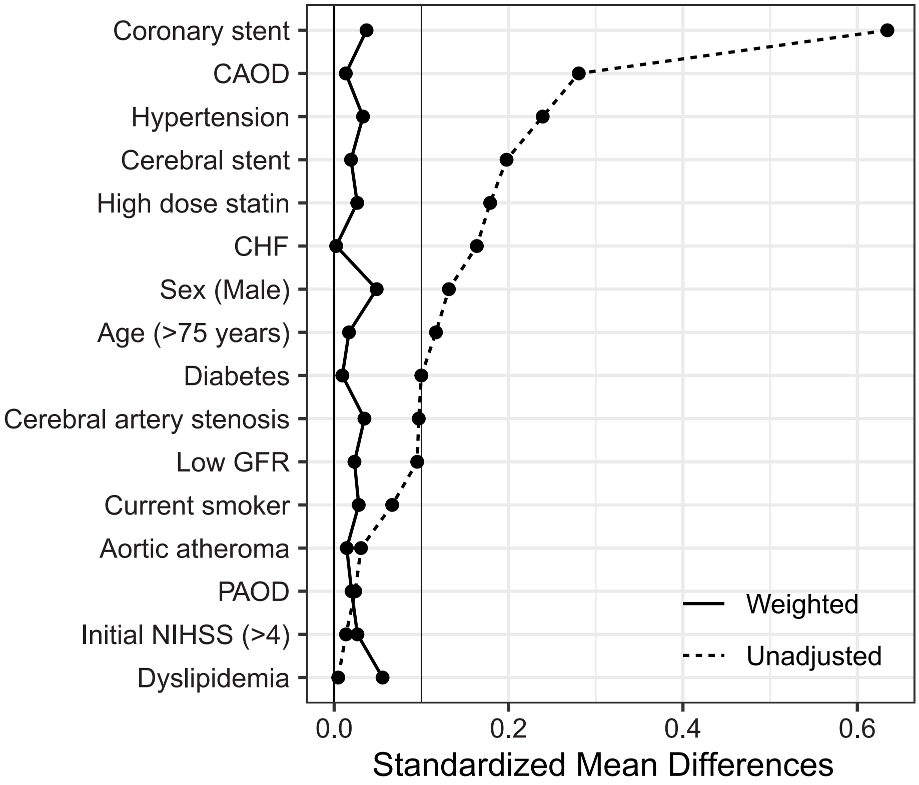

CAOD, coronary artery occlusive disease; CHF, chronic heart failure; GFR, glomerular filtration rate; PAOD, peripheral artery occlusive disease; NIHSS, NIH Stroke Scale
